# Sensitization of multidrug-resistant human cancer cells to Hsp90 inhibitors by down-regulation of SIRT1

**DOI:** 10.18632/oncotarget.5343

**Published:** 2015-09-25

**Authors:** Hak-Bong Kim, Su-Hoon Lee, Jee-Hyun Um, Won Keun Oh, Dong-Wan Kim, Chi-Dug Kang, Sun-Hee Kim

**Affiliations:** ^1^ Department of Biochemistry, Pusan National University School of Medicine, Yangsan 626-870, Korea; ^2^ Korea Mouse Metabolic Phenotyping Center, Lee Gil Ya Cancer and Diabetes Institute, Gachon University, Incheon 406-840, Korea; ^3^ Korea Bioactive Natural Material Bank, College of Pharmacy, Seoul National University, Seoul 151-818, Korea; ^4^ Department of Microbiology, College of Natural Sciences, Chang Won National University, Chang Won 641-773, Korea

**Keywords:** Hsp90 inhibitor, MDR, SIRT1, P-gp, Hsp70

## Abstract

The effectiveness of Hsp90 inhibitors as anticancer agents was limited in multidrug-resistant (MDR) human cancer cells due to induction of heat shock proteins (Hsps) such as Hsp70/Hsp27 and P-glycoprotein (P-gp)-mediated efflux. In the present study, we showed that resistance to Hsp90 inhibitors of MDR human cancer cells could be overcome with SIRT1 inhibition. SIRT1 knock-down or SIRT1 inhibitors (amurensin G and EX527) effectively suppressed the resistance to Hsp90 inhibitors (17-AAG and AUY922) in several MDR variants of human lymphoblastic leukemia and human breast cancer cell lines. SIRT1 inhibition down-regulated the expression of heat shock factor 1 (HSF1) and subsequently Hsps and facilitated Hsp90 multichaperone complex disruption via hyperacetylation of Hsp90/Hsp70. These findings were followed by acceleration of ubiquitin ligase CHIP-mediated mutant p53 (mut p53) degradation and subsequent down-regulation of P-gp in 17-AAG-treated MDR cancer cells expressing P-gp and mut p53 after inhibition of SIRT1. Therefore, combined treatment with Hsp90 inhibitor and SIRT1 inhibitor could be a more effective therapeutic approach for Hsp90 inhibitor-resistant MDR cells via down-regulation of HSF1/Hsps, mut p53 and P-gp.

## INTRODUCTION

Heat shock protein 90 (Hsp90) is a molecular chaperone whose association is required for the stability and function of numerous oncoproteins that promote the growth and/or survival of cancer cells, and therefore may serve as a therapeutic target for the treatment of cancer [1]. Several Hsp90 inhibitors such as 17-allylamino-17-demethoxy-geldanamycin (17-AAG), an ansamycin Hsp90 inhibitor, and NVP-AUY922 (hereafter called AUY922), a non-ansamycin Hsp90 inhibitor, have shown potent antitumor activity in a wide-range of malignancies [2–5]. However, it is known that treatment with Hsp90 inhibitors leads, through a negative feedback loop, to activation of the heat shock factor (HSF1), which causes transcriptional induction of Hsp70/Hsp27 that protect cancer cells from apoptosis and induce drug resistance [6, 7], offsetting their own anticancer activities. Although 17-AAG and AUY922 inhibit the function of Hsp90, they usually increase the heat shock proteins (Hsps) such as Hsp70/Hsp27, which reduce the Hsp90-targeted drug efficacy by inhibiting apoptosis signaling [8–10]. Indeed, resistance to 17-AAG is linked to the induction of Hsp70/Hsp27 and P-glycoprotein (P-gp)-mediated efflux [11–15]. The 17-AAG sensitivity was significantly increased by transfection of Hsp27 and/or Hsp70 siRNA in 17-AAG-resistant cells [14]. HSF1 also appears to regulate multiple pathways involved in expression of multidrug-resistant (MDR) phenotype in addition to induction of Hsps that leads to escape from chemotherapy-mediated cell killing. *MDR1* gene that encodes P-gp is known to contain heat shock elements in the promoter region, and HSF1 can activate *MDR1* expression in a stress-independent manner [16]. Therefore, silencing HSF1/Hsps could cause an increased sensitivity of MDR cells to Hsp90 inhibitor possibly by down-regulation of P-gp. HSF1 can also control the stability of mut p53 protein in human cancer cells. It has been known that naturally unfolded mutant p53 (mut p53) is an Hsp90 client protein and forms stable complex with Hsp90 multichaperone machinery [17]. Knockdown of HSF1 in mut p53 (+) cancer cells, which leads to down-regulation of Hsps, induces rapid destabilization of mut p53 [18]. As an oncoprotein, mut p53, a hallmark of almost 50% of human tumors, up-regulates the expression of *MDR1* gene and may confer upon tumor cells a selective survival advantage during chemotherapy [19]. Therefore, it is necessary to develop new therapeutics that can induce mut p53 protein degradation. It has been demonstrated that in the absence of Hsp90 activity, the less stable unfolded mut p53 protein preferentially associate in a complex with Hsp70 and CHIP (carboxyl terminus of Hsp70-interacting protein) ubiquitin ligase [17], which has a major role for in the degradation of unfolded mut p53, with little or no roles for CHIP in degrading wild-type p53 protein [20]. This CHIP-mediated degradation of mut p53 would suppress the expression of *MDR1*/P-gp in MDR cells.

Sirtuin 1 (SIRT1), a class III histone deacetylase (HDAC), is required for the activation and stability of HSF1, which in turn is responsible for Hsp70 induction [21]. Down-regulation of SIRT1 resulted in a release of the acetylated HSF1 from its cognate promoter elements [22]. However, it is not known whether SIRT1 inhibition may modulate the effect of Hsp90 inhibitors, which offset their own effect on anticancer activity through suppression of HSF1/Hsps and degradation of mut p53.

In the present study, we demonstrated that SIRT1 inhibitor sensitized MDR cells to Hsp90 inhibitors via down-regulation of HSF1/Hsps, mut p53 and P-gp consequently overcoming resistance of MDR cells to Hsp90 inhibitors.

## RESULTS

### Resistance of MDR cells with high level of P-gp to Hsp90 inhibitors

**Figure 1:**
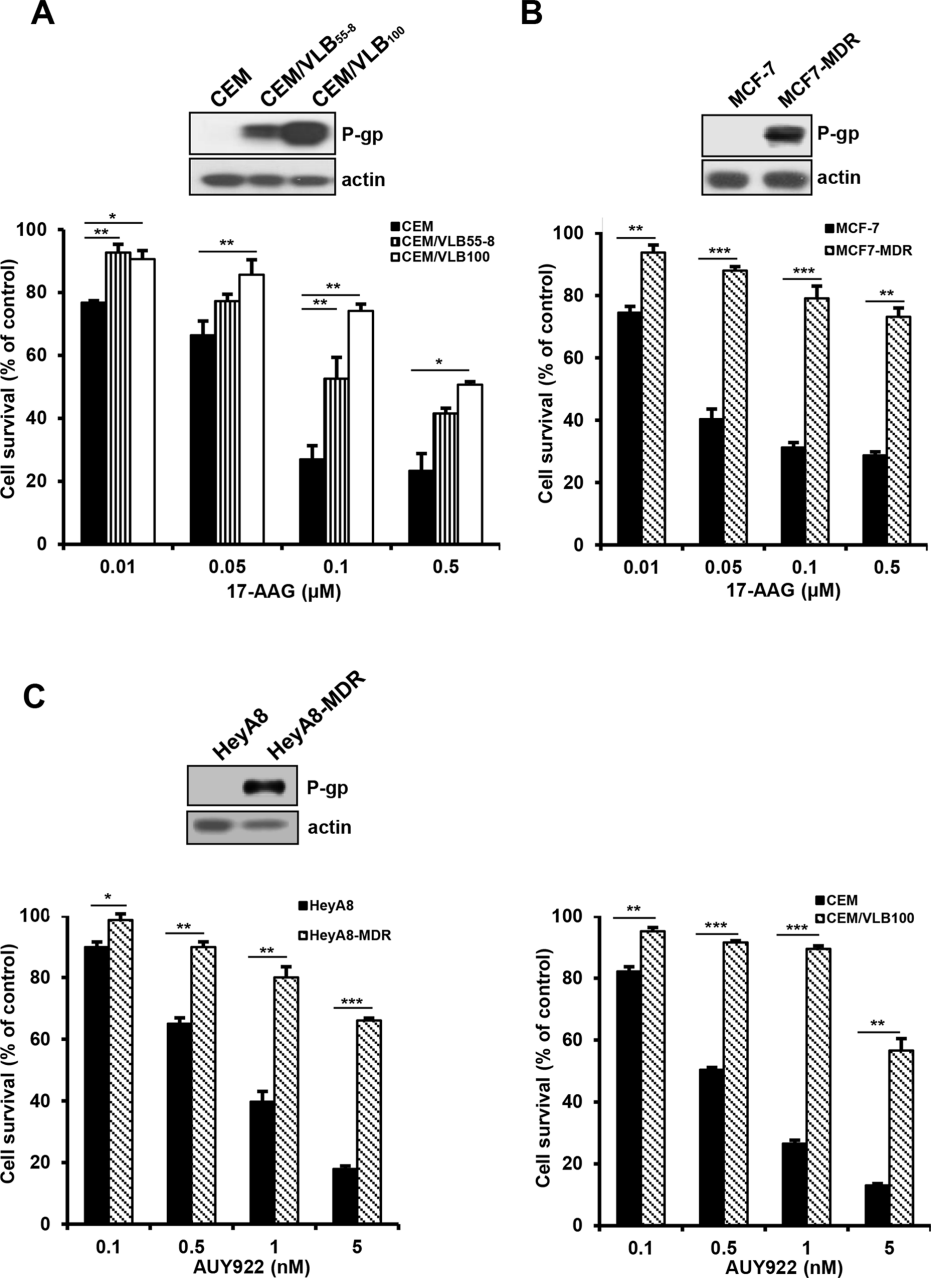
Differential cytotoxic effect of Hsp90 inhibitors in a range of cancer cell lines and their MDR variants Human leukemic CEM **A.** human breast cancer MCF-7 **B.** and human ovarian cancer HeyA8 cell lines **C.** and their MDR variants overexpressing P-gp were treated with serial concentrations of 17-AAG or AUY922. Percentage of cell survival was determined after 96 h of incubation using MTT assay. Results are the means ± SEs of three experiments. *p < 0.05, **p < 0.01 and ***p < 0.001. Western blot analysis was performed to monitor the protein levels of P-gp and β-actin (actin) is used as a loading control of all cell lines.

**Figure 2:**
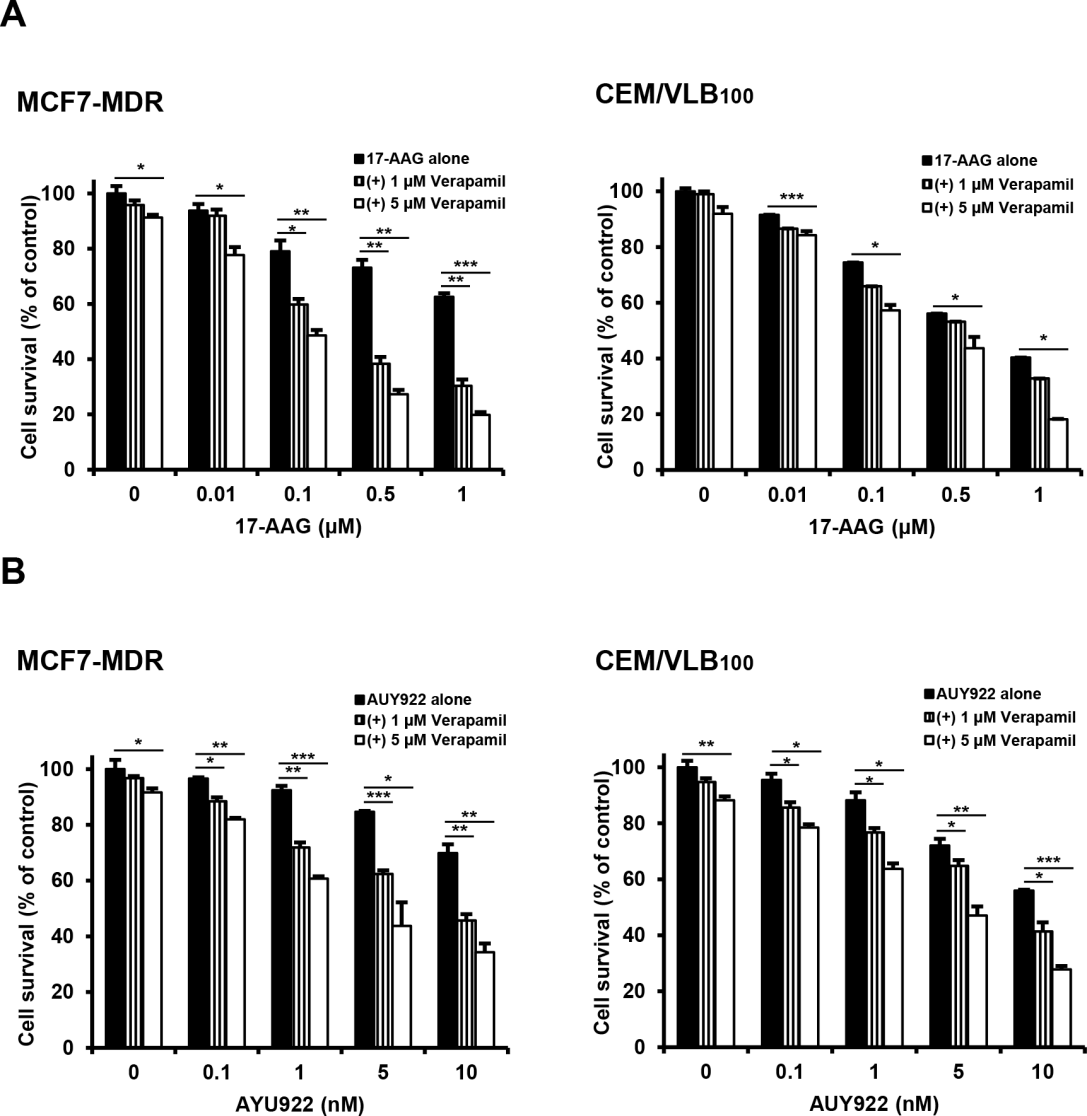
Potentiation of cytotoxicity of Hsp90 inhibitors in MDR cells by P-gp inhibitor verapamil MCF-7/MDR or CEM/VBL_100_ cells were treated with serial doses of 17-AAG **A.** or AUY922 **B.** in the presence or absence of verapamil (1- and 5 μM). Percentage of cell survival was determined after 96 h of incubation using MTT assay. Results are the means ± SEs of three experiments. *p < 0.05, **p < 0.01 and ***p < 0.001.

To compare the susceptibility of Hsp90 inhibitor-mediated cytotoxicity between CCRF-CEM (hereafter called CEM) human leukemia cells, MCF-7 human breast cancer cells and HeyA8 human ovarian cancer cells, and their respective MDR variants, they were treated with increasing doses of Hsp90 inhibitors including 17-AAG or another structurally unrelated AUY922, and then the cytotoxicity was determined by MTT assay. CEM/VLB_55–8_ and CEM/VLB_100_, MDR variants of CEM cells, expressing moderate and high levels of P-gp, respectively, exhibited a marked resistance to 17-AAG compared with their parental CEM cells. Similar results were observed in between MCF7-MDR, a MDR variant of MCF-7, with high level of P-gp and their parental MCF-7 cells (Figure 1A and 1B). In addition, HeyA8-MDR, a MDR variant of HeyA8, and CEM/VLB_100_ cells were highly resistant to AUY922 when compared to their parental counterparts (Figure 1C). MCF7-MDR cells also were highly resistant to AUY922 (data not shown). These results suggest that P-gp expression might contribute to resistance of MDR cells to Hsp90 inhibitors. To determine whether the resistance to Hsp90 inhibitors is dependent on the P-gp in MDR cells, MCF7-MDR and CEM/VLB_100_ cells were treated with 17-AAG in the presence of verapamil, a pharmacologic inhibitor of P-gp. The susceptibility of both MDR cells to 17-AAG was increased by treatment with verapamil dose-dependently (Figure 2A). Similar results were obtained in AUY922-treated MDR cells. Verapamil potentiated the cytotoxicity of AUY922 on MCF7-MDR and CEM/VLB_100_ cells (Figure 2B). Therefore, it could be suggested that P-gp expression might contribute at least in part to the resistance of MDR cells to Hsp90 inhibitors.

### Involvement of 17-AAG-mediated activation of HSF1 in resistance of MDR cells to 17-AAG

**Figure 3:**
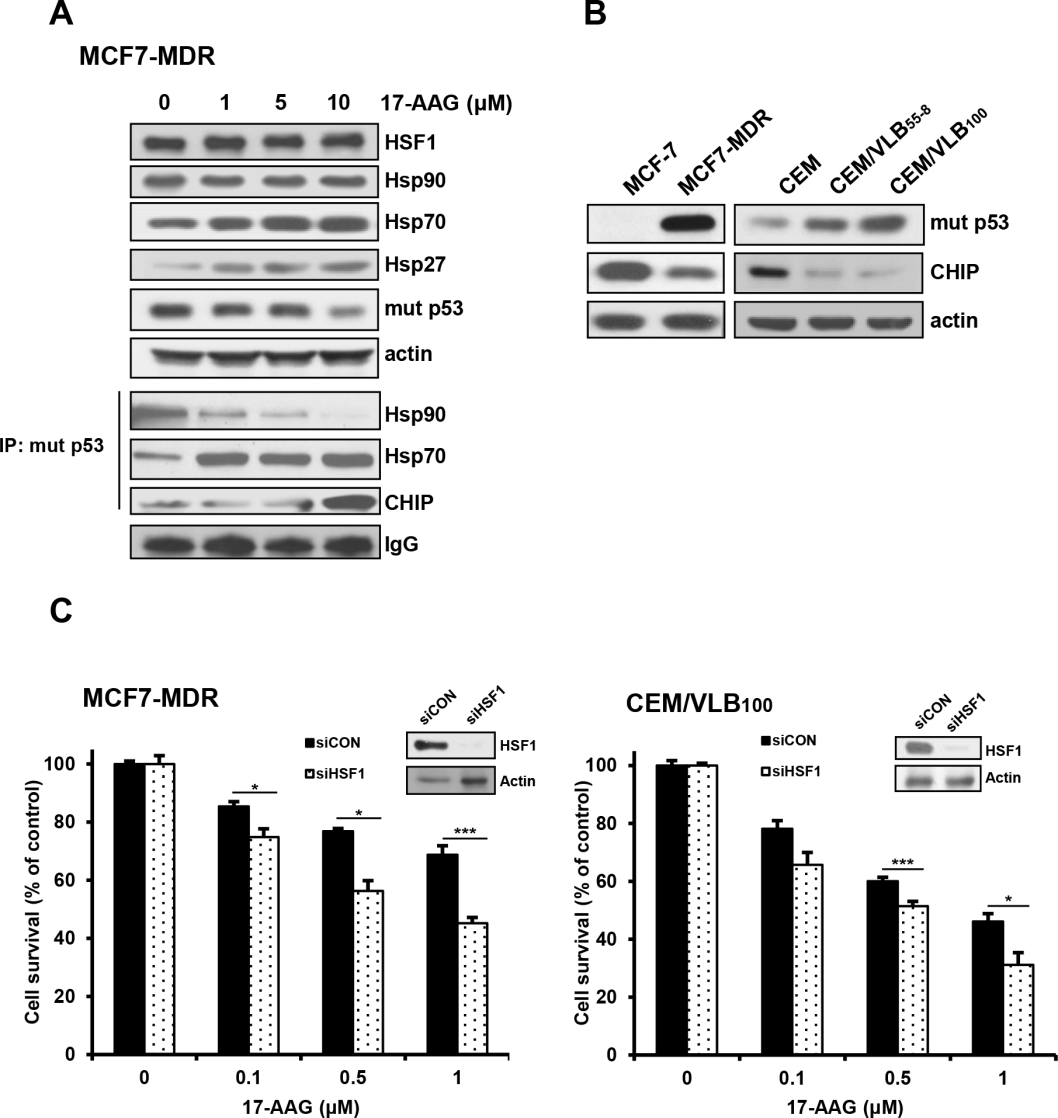
17-AAG-mediated activation of HSF1/Hsps in MDR cells and enhanced cytotoxicity of MDR cells to 17-AAG by HSF1 inhibition **A.** The changed levels of HSF1/Hsps and mut p53 in MCF7-MDR cells treated with serial doses of 17-AAG were determined by western blot analysis (upper panels). Cell lysates of MCF7-MDR cells treated with 17-AAG were immunoprecipitated with anti-p53 antibody and immunoblotted with anti-Hsp90, -Hsp70 and -CHIP. IgG was used as an internal control for the immunoprecipitation (lower panels). **B.** The basal protein levels of mut p53 and CHIP in MCF-7 and CEM cells and their MDR counterparts. **C.** MCF-7/MDR or CEM/VBL_100_ cells were transfected with 20 nM HSF1 siRNA (siHSF1) or scrambled siRNA for control (siCON). After 48 h of transfection, the cells were treated with serial concentrations of 17-AAG. Percentage of cell survival was determined after 96 h of incubation using MTT assay. Each bar represents the mean ± S.D. of triplicate experiments. *p < 0.05 and ***p < 0.001.

It has been shown that inhibition of Hsp90 function shifts the chaperone association of client proteins such as mut p53 from Hsp90 to Hsp70, resulting in their proteasomal degradation [23], and dissociates the Hsp90-HSF1 complex, causing the activation of HSF1 and consequently HSF1-mediated induction of Hsps [6]. To demonstrate the involvement of HSF1-mediated induction of Hsps in resistance of MDR cells to Hsp90 inhibitor, activation of HSF1 and the levels of Hsps were determined after treatment of MCF-7 MDR cells with 17-AAG. The activation of HSF1 evidenced by an electrophoretic mobility shift and levels of Hsp70/Hsp27 were increased by treatment with 17-AAG, dose-dependently. These results were accompanied with the dissociation of mut p53 and Hsp90, and subsequent association of mut p53 with Hsp70 and ubiquitin ligase CHIP, which resulted in a decrease in mut p53 level (Figure 3A), indicating the HSF1-mediated induction of Hsps and decrease of mut p53 possibly through formation of complex with Hsp70 and CHIP after treatment with Hsp90 inhibitor. Interestingly, this reverse correlation between the levels of mut p53 and CHIP was demonstrated in various MDR variants, including MCF7-MDR, CEM/VLB_55–8_ and CEM/VLB_100_, in which expression of mut p53 protein was increased, and the level of ubiquitin ligase CHIP that targets ubiquitination and degradation of mut p53 was conversely correlated with the level of mut p53 when compared with their parental cells, suggesting stabilization of mut p53 by down-regulation of CHIP in MDR cells (Figure 3B). Then, it was determined if inhibition of HSF1 could increase the susceptibility of MDR cells to 17-AAG. After knockdown of HSF1 with siRNA in MCF7-MDR and CEM/VLB_100_ cells, the sensitivity of 17-AAG was significantly increased in both MDR cells (Figure 3C). These results suggest that inhibition of HSF1 activity as well as P-gp may be useful to suppress the resistance of MDR cells to HSP90 inhibitors.

### Down-regulation of HSF1/Hsps and mut p53 and decreased formation of Hsp90 multichaperone complex by SIRT1 inhibition via hyperacetylation of Hsp90/Hsp70 and mut p53

**Figure 4:**
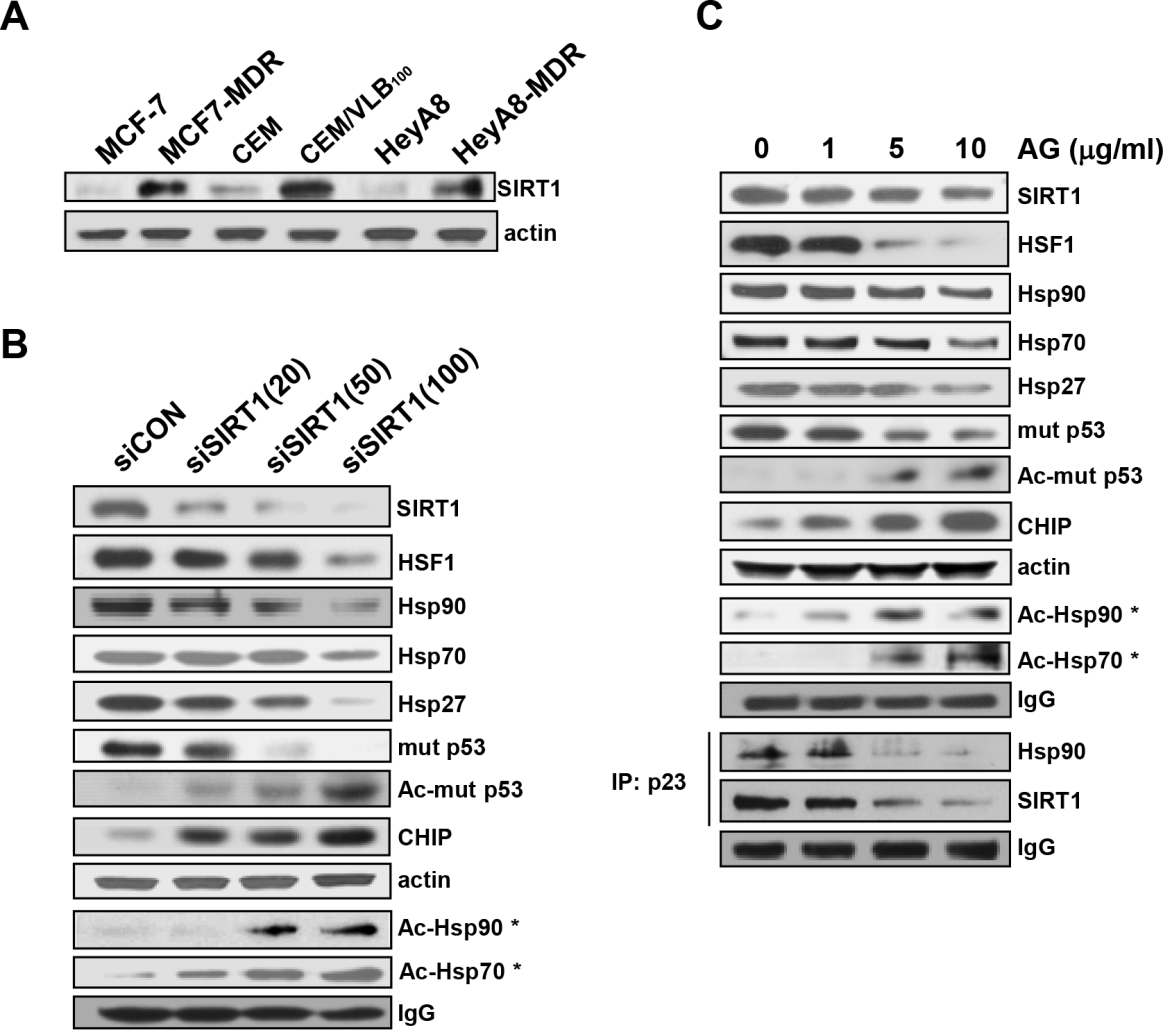
Down-regulation of HSF1/Hsps and mut p53 and decreased formation of Hsp90 chaperone complex by SIRT1 inhibition **A.** The protein level of SIRT1 in MCF7-MDR, CEM/VBL_100_ and HeyA8-MDR cells and their parental cells was determined by western blot analysis. MCF7-MDR cells were transfected with the indicated dose of 20 nM scrambled siRNA for control (siCON) or 20∼100 nM SIRT1 siRNA (siSIRT) for 48 h **B.** or treated with the indicated dose of amurensin G (AG) for 24 h **C.** and western blot analysis was performed to monitor protein levels of HSF1, Hsps, CHIP and total and acetylated mut p53. For determination of acetylated Hsp90 (Ac-Hsp90*) or Hsp70 (Ac-Hsp70*), cell lysates from (A) or (B) were immunoprecipitated with anti-pan-AcK antibody and immunoblotted with anti-Hsp90 or Hsp70 antibody. In addition, cell lysates treated with AG were immunoprecipitated with anti-p23 antibody and immunoblotted with anti-Hsp90 or -SIRT1 antibody (B, lower panels). The level of IgG was used as a loading control.

Since it has been shown that up-regulation of SIRT1 is correlated with P-gp-mediated MDR phenotype [24], and down-regulation of SIRT1 accelerated the attenuation of the heat shock response by inactivation of HSF1 through increased acetylation [21], we first examined the level of SIRT1 in MDR cells. Up-regulation of SIRT1 was observed in MCF7-MDR, CEM/VLB_100_ and HeyA8-MDR cells compared with their parental cells (Figure 4A). We next determined whether inhibition of SIRT1 could suppress HSF1-dependent pathways in MDR cells (Figure 4B). When SIRT1 was knock-downed in MCF7-MDR cells with increasing amounts of SIRT1 siRNA, the levels of HSF1 and Hsps were down-regulated in the MDR cells. In addition, the acetylation of Hsp90 and Hsp70, which disrupt Hsp90 chaperone function [25], was increased by knockdown of SIRT1. This result was followed by hyperacetylation and decrease of mut p53 and increase of CHIP. Similar results were observed in MCF7-MDR cells treated with gradual doses of amurensin G, a natural SIRT1 inhibitor (Figure 4C, upper panels). Since SIRT1 inhibition resulted in inactivation of HSF1 through hyperacetylation [21], and mut p53 can activate HSF1 directly and indirectly [26], our results suggest the possibility that depletion of SIRT1 lead to inhibition of HSF1-dependent pathway through hyperacetylation of Hsp90/Hsp70 and mut p53. In addition, treatment of MCF-7/MDR cells with amurensin G led to a decreased binding of cochaperone p23 to Hsp90 and SIRT1 (Figure 4B, lower panels). Since the p23 locks Hsp90 in a conformational state with a high affinity for client proteins, promoting the folding and stabilization of client proteins [27], our results suggest that SIRT1 inhibition may promote degradation of Hsp90 client protein such as mut p53 through the decreased formation of Hsp90/SIRT1/p23 complex.

### Suppression of 17-AAG-mediated activation of HSF1-dependent pathway and enhancement of 17-AAG-induced apoptosis by SIRT1 inhibition

**Figure 5:**
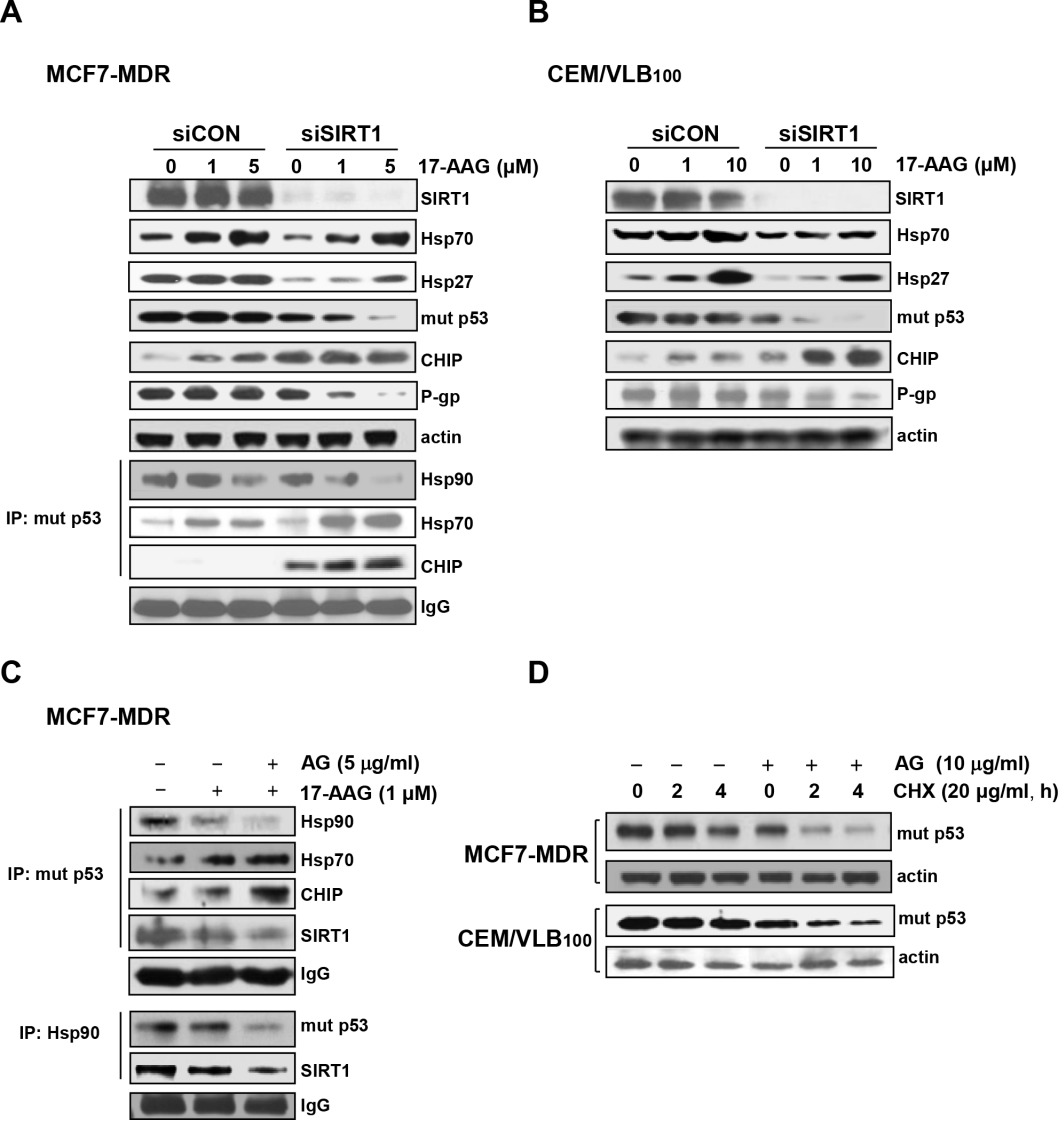
Suppression of 17-AAG-mediated activation of HSF1-dependent pathway and Hsp90 chaperone complex formation of by SIRT1 inhibition **A.** and **B.** MCF7-MDR or CEM/VBL_100_ cells transfected with of 20 nM siCON or siSIRT1 for 48 h were additionally treated with indicated doses of 17-AAG for 24 h, and whole cell lysates of them were analysed by immunoblotting to measure changed expression of SIRT1, Hsp70/Hsp27, mut p53, CHIP and P-gp (upper panels). The changed binding of mut p53 with Hsp90 and CHIP in SIRT1-depleted MCF7-MDR cells was determined by immunoprecipitation with anti-p53 and immunoblotting with anti-Hsp90, Hsp70 and CHIP (left lower panels). **C.** Cell lysates from MCF7-MDR cells treated with 17-AAG (1 μM) in the presence or absence of amurensin G (5 μg/ml) were immunoprecipitated with anti-p53 or Hsp90 and immunoblotted with anti-Hsp90, Hsp70, CHIP, SIRT1 or mut p53, respectively. **D.** MCF7-MDR or CEM/VBL_100_ cells were treated with cycloheximide (CHX, 20 μg/ml) in the presence or absence of AG (10 μg/ml). Then the MDR variants were and harvested at different time points (2 or 4 h) and, the changed level of mut p53 was determined by western blot analysis.

**Figure 6:**
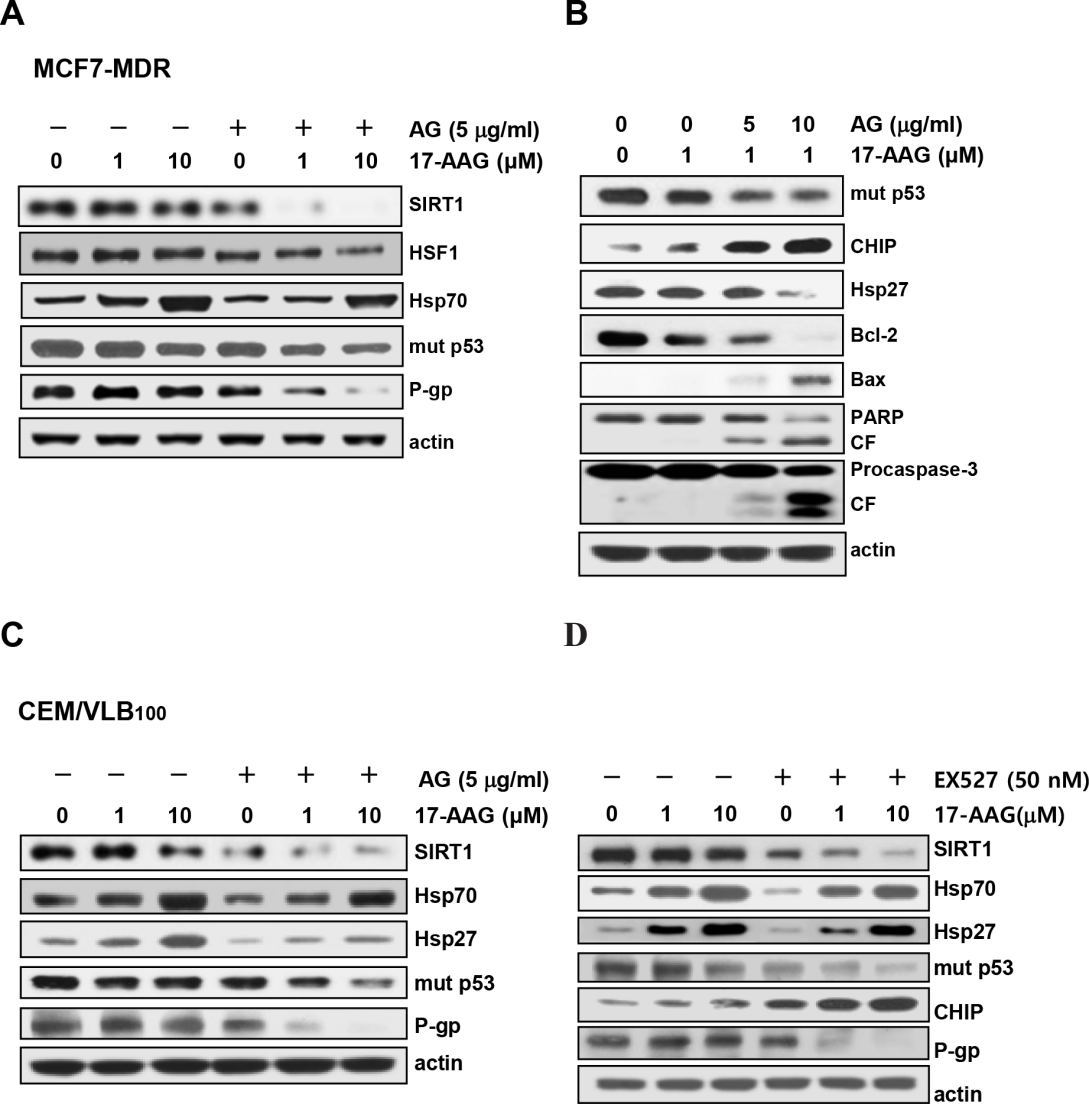
Potentiation of 17-AAG-mediated down-regulation of Hsps and mut p53/P-gp and activation of pro-apoptotic cascade by SIRT1 inhibitor **A.** MCF7-MDR cells were pretreated with AG (5 μg/ml) for 6 h followed by 17-AAG (1 or 10 μM) for additional 24 h. Western blot analysis was performed to determine changed levels of SIRT1, HSF1/Hsp70, mut p53 and P-gp. **B.** MCF7-MDR cells were pretreated with AG (5 or 10 μg/ml) for 6 h followed by 17-AAG (1 μM) for additional 24 h, and the changed levels of mut p53, CHIP, Hsp27, Bcl-2, and Bax and the activation of PARP and procaspase-3 were determined by western blot analysis (right panels). CF, cleavage fragment. CEM/VBL_100_ cells were pretreated with AG (5 μg/ml) **C.** or EX527 (50 nM) **D.** for 6 h followed by 17-AAG (1 or 10 μM) for additional 24 h western blot analysis was performed to determine changed levels of SIRT1, Hsp70/Hsp27, mut p53, CHIP and P-gp.

**Figure 7:**
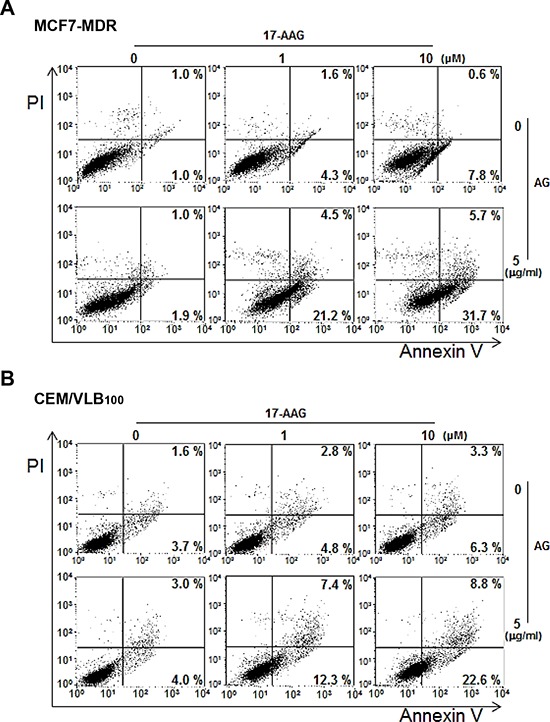
Enhanced apoptosis with combination 17-AAG/amurensin G treatment in MDR cells MCF7-MDR **A.** or CEM/VBL_100_ cells **B.** were treated with 17-AAG (1 or 10 μM) and/or amurensin G (AG, 5 μg/ml) for 24 h, and the percentages of early and late apoptotic cells were quantified by FACS for Annexin-V and PI staining. The bottom right quadrant of each dot plot represents early apoptotic cells (annexin V positive/PI negative). The upper right quadrant represents late apoptotic cells (annexin V positive/PI positive).

Since it has been reported that HSF1-dependent induction of Hsps occurs frequently in response to Hsp90 inhibitor, and it attenuates Hsp90 inhibitor-induced cytotoxicity [11], and our data showed that depletion of SIRT1 lead to inhibition of HSF1-dependent pathway, we determined whether inhibition of SIRT1 could suppress 17-AAG-mediated activation of HSF1-dependent pathway in MCF7-MDR and CEM/VBL_100_ cells. SIRT1 knockdown attenuated 17-AAG-mediated induction of Hsp70/Hsp27, and reinforced down-regulation of mut p53 and up-regulation of CHIP in both MDR cells. In addition, the level of P-gp was remarkably down-regulated after knockdown of SIRT1 in the presence of 17-AAG (Figure 5A and 5B). We further investigated the interaction of mut p53 with Hsp90/Hsp70 and CHIP in 17-AAG-treated MCF7-MDR cells after knockdown of SIRT1 using co-immunoprecipitation assay. The 17-AAG-mediated dissociation of mut p53 and Hsp90, and association of mut p53 with Hsp70 and CHIP were reinforced by SIRT1 knockdown (Figure 5A, lower panels) or by amurensin G in MCF7-MDR cells (Figure 5C, upper panels). When the reverse co-immunoprecipitation assay was performed with an anti-Hsp90, binding of Hsp90 to mut p53 and SIRT1 was significantly reduced by co-treatment of MCF7-MDR cells with 17-AAG and amurensin G than with 17-AAG alone (Figure 5C, lower panels). To determine whether amurensin G down-regulated mut p53 at the posttranslational level, the level of mut p53 protein in the both MDR cells was determined in the presence of cycloheximide (CHX), a protein synthesis inhibitor after treatment with amurensin G (Figure 5D). We found that mut p53 protein started to decrease at 4 h after treatment with CHX alone but markedly decreased at 2 h after co-treatment of both MDR cells with CHX and amurensin G, suggesting that SIRT1 inhibition reduced the half-life of mut p53 possibly through degradation of the protein. We observed consistent results with amurensin G in both MDR cells, including MCF7-MDR and CEM/VBL_100_ cells, in comparison with siSIRT1 (Figure 6A and 6C). In addition, treatment with amurensin G reinforced down-regulation of Bcl-2, up-regulation of Bax, and activation of caspase-3-mediated PARP cleavage in MCF7-MDR cells in the presence of 17-AAG (Figure 6B). Similarly, EX527, a SIRT1-specific inhibitor, suppressed 17-AAG-induced Hsp70/Hsp27 expression and accelerated 17-AAG-induced down-regulation of mut p53 and P-gp and up-regulation of CHIP in CEM/VBL_100_ cells (Figure 6D). We therefore determined whether amurensin G can enhance susceptibility of MDR cells to 17-AAG-induced apoptosis. Co-treatment with amurensin G enhanced 17-AAG-induced apoptosis in both MCF7-MDR and CEM/VBL_100_ cells (Figure 7). Similarly, potentiation of 17-AAG-induced apoptosis by amurensin G was observed in HeyA8-MDR cells (data not shown). These results suggest that SIRT1 inhibition would be used to sensitize MDR cells to 17-AAG-induced apoptosis through suppression of activation of HSF1-dependent pathway and down-regulation of P-gp expression.

### Suppression of P-gp-mediated efflux activity in MDR cells by SIRT1 inhibitors

**Figure 8:**
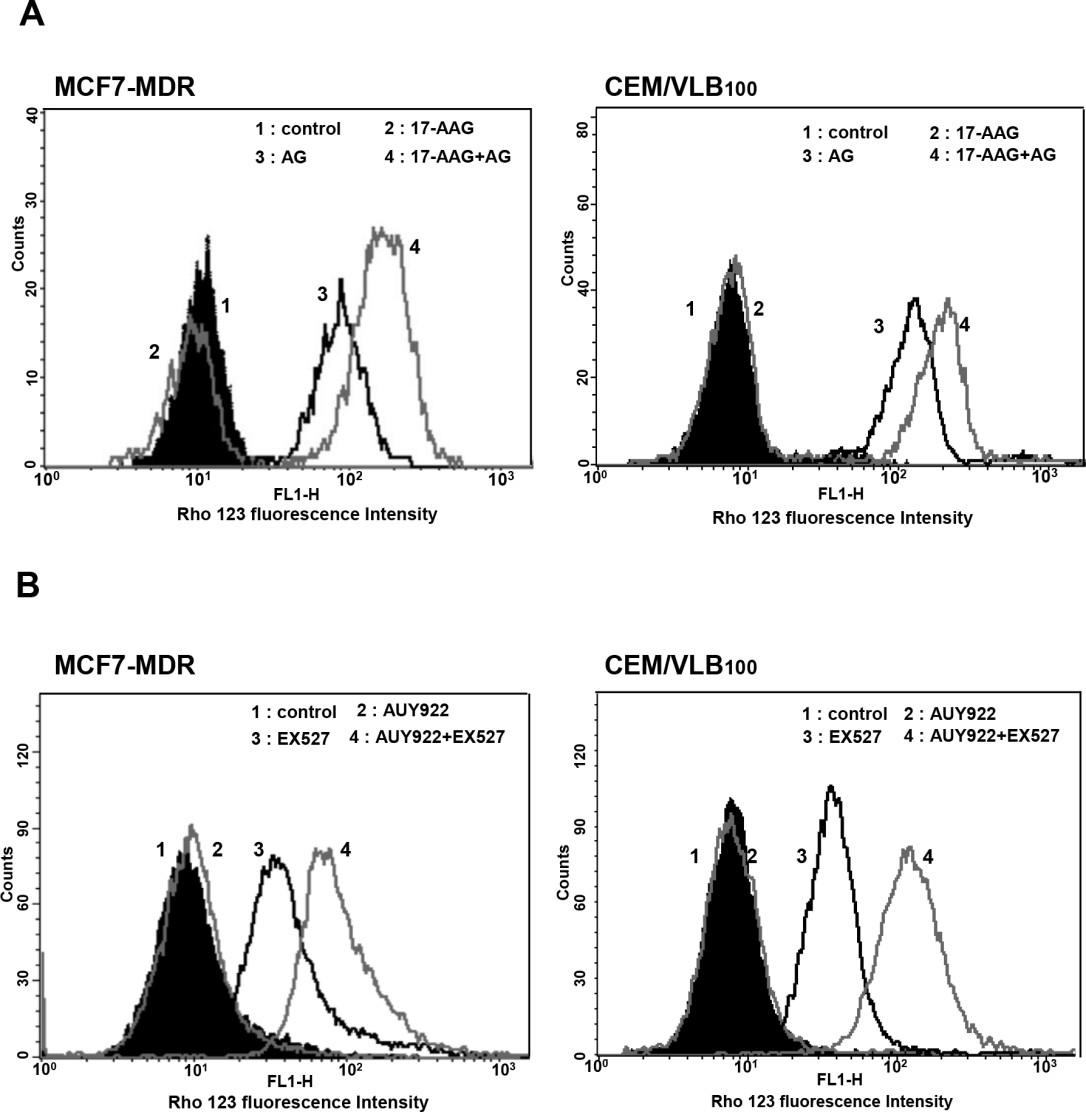
Effect of SIRT1 inhibitors on P-gp-mediated efflux activity of Hsp90 inhibitors in MDR cells **A.** Cell suspension isolated from MCF7-MDR or CEM/VBL_100_ cells treated with 17-AAG (5 μM) in the presence or absence of AG (5 μg/ml) or for 12 h. **B.** Cell suspension of MCF7-MDR or CEM/VBL_100_ cells treated with AUY922 (0.01 μM) in the presence or absence of EX527 (50 nM) for 12 h. And then these are incubated with Rho123 and further incubated at 37°C for 4 h to allow P-gp-mediated efflux. Cellular fluorescence was analyzed immediately by using flow cytometer.

We then evaluated whether the down-regulation of P-gp by combined treatment with Hsp90 inhibitor and SIRT1 inhibitor would lead to the reduction of P-gp-mediated efflux activity in MDR cells using a flow cytometric functional efflux assay based on the extrusion of rhodamine 123 (Rho 123), a fluorescent substrate for P-gp. Amurensin G or EX527 significantly suppressed efflux of Rho 123 and consequently increased the intracellular level of fluorescence, and combined treatment of amurensin G or EX527 with 17-AAG suppressed further the efflux of Rho 123 in MCF7-MDR and CEM/VBL_100_ cells, meanwhile 17-AAG, a substrate of P-gp, did not show any effect on Rho 123 efflux at a concentration used (Figure 8A). These results were consistent with the decreased level of P-gp after combined treatment of amurensin G with 17-AAG in both MDR cells. In addition, we observed also very similar results with other set of Hsp90 inhibitor, AUY922, and SIRT1 inhibitor, EX527, in both MDR cells (Figure 8B). EX527 also suppressed efflux of Rho 123 and consequently increased the intracellular level of fluorescence, and combined treatment of EX527 and AUY922 suppressed further the efflux of Rho 123 in MCF7-MDR and CEM/VBL_100_ cells, while AUY922 did not show any effect on Rho 123 efflux at a concentration used, indicating that SIRT1 inhibition could suppress the resistance of MDR cancer cells to AUY922 as well as 17-AAG through suppression of P-gp-mediated efflux. Therefore, these results suggest that SIRT1 inhibitor is highly effective to increase an efficacy of Hsp90 inhibitors through down-regulation of P-gp and consequently to sensitize MDR cells to Hsp90 inhibitors.

### Sensitization of MDR cells to 17-AAG by SIRT1 inhibition

**Figure 9:**
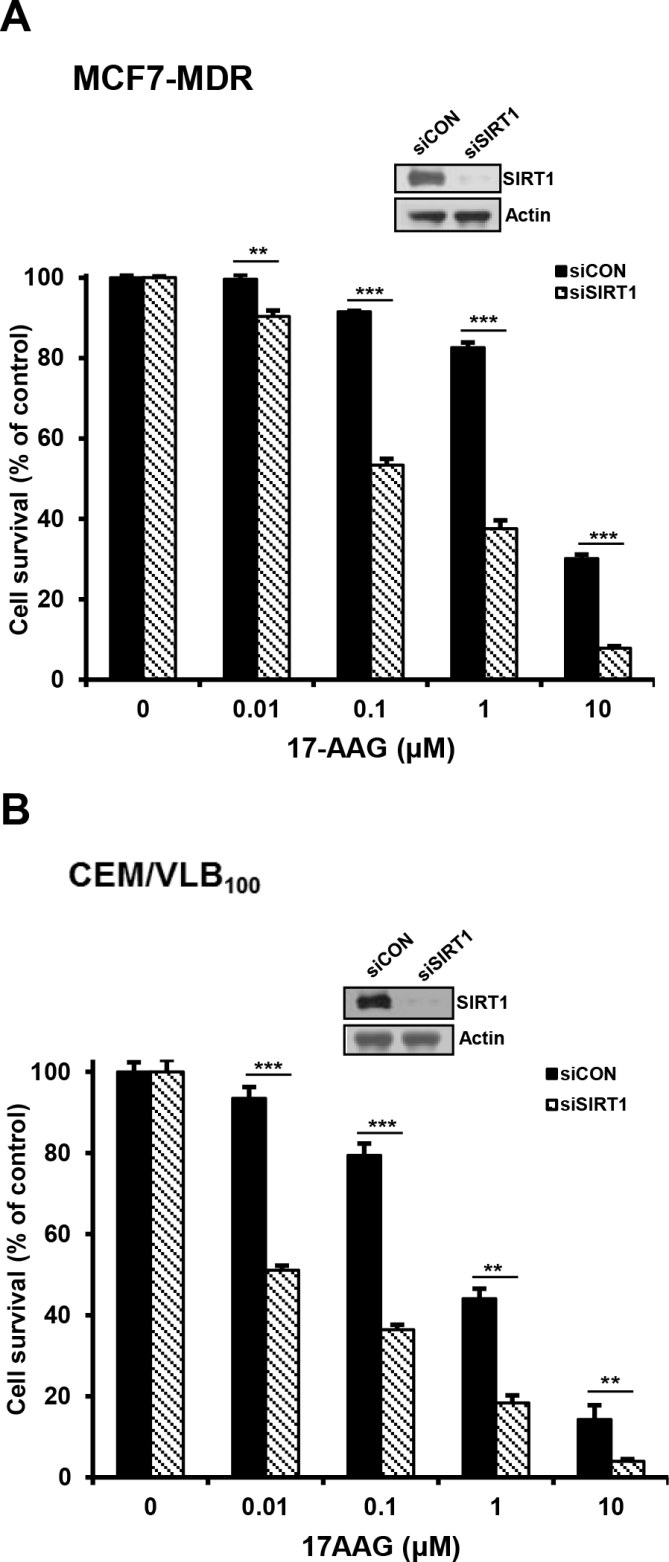
Sensitization of MDR cells to 17-AAG by SIRT1 depletion **A.** and **B.** MCF-7/MDR or CEM/VBL_100_ cells were transfected with 20 nM SIRT1 siRNA (siSIRT1) or scrambled siRNA for control (siCON). After 48 h of transfection, changed level of SIRT1 was determined by western blot analysis, and the transfectants were treated with serial concentrations of 17-AAG. Percentage of cell survival was determined after 96 h of incubation using MTT assay. Each bar represents the mean ± S.D. of triplicate experiments. **p < 0.01 and ***p < 0.001.

**Figure 10:**
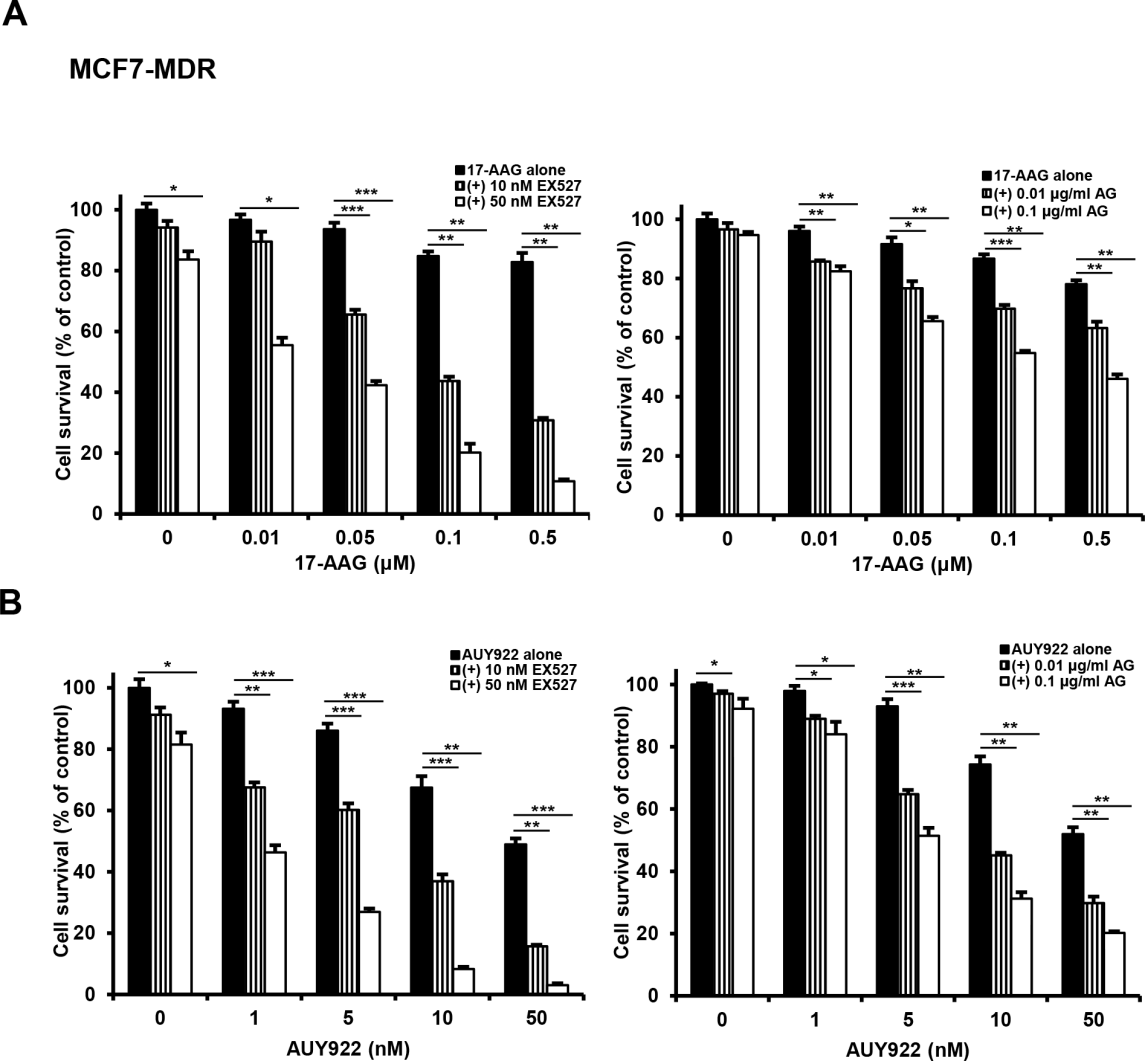
Potentiation of cytotoxicity of Hsp90 inhibitors in MCF7-MDR cells by SIRT1 inhibitors MCF7-MDR cells were treated with serial doses of 17-AAG **A.** or AUY922 **B.** in the presence or absence of amurensin G (AG; 0.01- and 0.1 μg/ml) or EX527 (10- and 50 nM). Percentage of cell survival was determined after 96 h of incubation using MTT assay. Results are the means  ±  SEs of three experiments. *p < 0.05, **p < 0.01 and ***p < 0.001.

**Figure 11:**
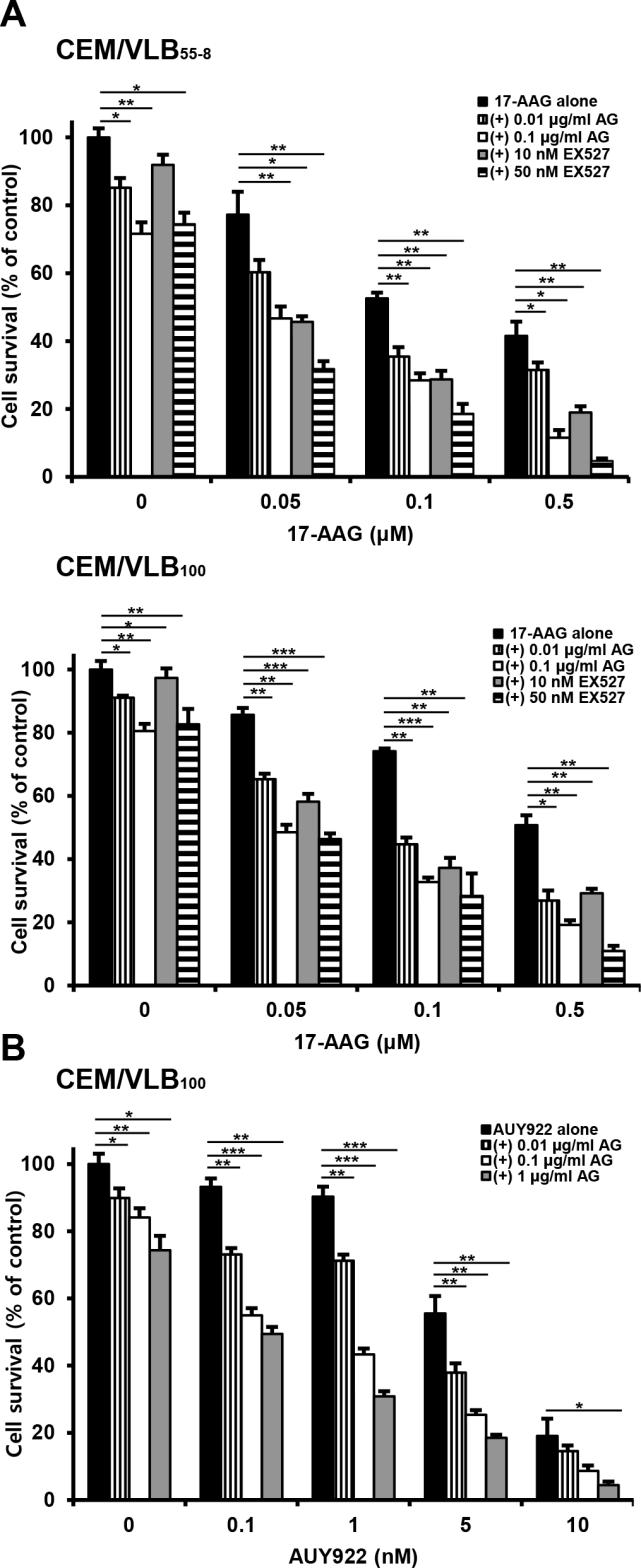
Effect of SIRT1 inhibitors on cytotoxicity of Hsp90 inhibitors in MDR variants of CEM cells **A.** CEM/VBL_55–8_ and CEM/VBL_100_ cells, variants of CEM cells were treated with serial doses of 17-AAG in the presence or absence of amurensin G (AG; 0.01- and 0.1 μg/ml) or EX527 (10- and 50 nM). **B.** CEM/VBL_100_ cells were treated with serial doses of AUY 922 in the presence or absence of amurensin G (AG; 0.01∼1 μg/ml) Percentage of cell survival was determined after 96 h of incubation using MTT assay. Results are the means ± SEs of three experiments. *p < 0.05, **p < 0.01 and ***p < 0.001.

Since SIRT1 inhibition suppressed activation of HSF1-dependent pathway and P-gp-mediated efflux activity sin 17-AAG-treated MDR cells, it was determined if SIRT1 inhibition could sensitize MDR cells to 17-AAG. When MCF7-MDR and CEM/VLB_100_ cells were treated with 17-AAG after SIRT1 knock-down, the sensitivity of the MDR cells to 17-AAG was significantly enhanced (Figure 9A and 9B). These results indicate that SIRT1 inhibition can enhance the susceptibility of MDR cells to 17-AAG. To examine further the potential relationship between SIRT1 activity and sensitivity to Hsp90 inhibitor in MDR cells, we tested the combination effect of Hsp90 inhibitor with SIRT1 inhibitors in MCF7-MDR cells. SIRT1 inhibitors including EX527 and amurensin G could increase the susceptibility of MCF7-MDR cells to Hsp90 inhibitors including 17-AAG and AUY922 (Figure 10A and 10B). The CI values were lower than 0.5 at all concentrations, indicating a synergistic effect of combination of both agents. The combination effect of Hsp90 inhibitor and SIRT1 inhibitor was also observed in MDR variants of CEM cells, including CEM/VLB_55–8_ and CEM/VLB_100_ cells (Figure 11). When CEM/VLB_55–8_ and CEM/VLB_100_ cells were co-treated with EX527 or amurensin G with 17-AAG, SIRT1 inhibitors enhanced cytotoxicity of 17-AAG synergistically in both MDR cells with CIs ranging from 0.1 to 0.8 at all concentrations of both agents (Figure 11A). In addition, amurensin G synergistically enhanced cytotoxicity of AUY 922 in CEM/VLB_100_ cells (Figure 11B). Similarly, the susceptibility of HeyA8-MDR cells to Hsp90 inhibitor was enhanced by SIRT1 inhibitors (data not shown). These results suggested that SIRT1 inhibition would sensitize MDR cells to Hsp90 inhibitors, possibly through suppression of activation of HSF1-dependent pathway and P-gp-mediated efflux activity.

## DISCUSSION

Despite their efficacy, the application of Hsp90 inhibitors has significant drawbacks since they stimulate the heat shock response leading to an increase in the expression of Hsps such as Hsp70 and Hsp27, which can mediate an evasion of apoptosis [15]. Subsequently, it is becoming increasingly evident that the combinatorial targeting of Hsps could be a more effective therapeutic approach. In addition, P-gp-mediated efflux of Hsp90 inhibitors limited their effectiveness. We found that inhibition of SIRT1 enhanced susceptibility of various human MDR cancer cells to Hsp90 inhibitors, possibly through down-regulation of Hsp70/Hsp27 and P-gp and reduction of P-gp-mediated efflux activity, suggesting that Hsp90 inhibitors would exert effective therapeutic activity on MDR cells in combination with SIRT1 inhibitors.

HSF1 may play an important role in tumor initiation, development and maintenance, and activation of HSF1 contributes to resistance to Hsp90 inhibitors [15]. Inhibition of Hsp90 leads to disruption of regulatory complexes of Hsp90 with HSF1, thereby causing HSF1-mediated induction of cytoprotective Hsp70/Hsp27. Activation of HSF1 also induces expression of *MDR1* gene and induces a MDR phenotype that allows escape of cancer cells from chemotherapy-mediated cell killing [16]. It has been demonstrated that both activity and level of HSF1 are positively regulated by SIRT1 [21, 28] and mut p53 [26]. In our study, mut p53 level was decreased by SIRT1 inhibition. Therefore, we suggest that SIRT1 inhibition down-regulate the activity and level of HSF1 and subsequently Hsps, and facilitated Hsp90 multichaperone complex disruption via hyperacetylation of Hsp90/Hsp70 as well. Actually, the sensitivity of 17-AAG was significantly increased after knockdown of HSF1 in Figure 3. Therefore, it is plausible that down-regulation of SIRT1 would prevent 17-AAG-mediated induction of HSF1, Hsp70 and P-gp, and consequently sensitize cancer cells to 17-AAG. Our results showed that SIRT1 inhibition resulted in suppression of 17-AAG-mediated HSF1 activation and Hsp70/Hsp27 induction in MDR cells, and this might be one of the mechanisms of the combination effect of Hsp90 inhibitor and SIRT1 inhibitor. Hsp90 is also responsible for the stability and function of mut p53 [17], which can up-regulate *MDR1* gene expression [19]. In addition, ubiquitin ligase CHIP is involved in degradation of mut p53, and the functional inactivation of CHIP is a cause of aberrant stabilization of mut p53 in cancer [20]. These relationships were demonstrated in various MDR cells, in which mut p53 was up-regulated, and CHIP down-regulated. These results might be responsible for an increased level of P-gp in MDR cells. The increased level of P-gp seemed to be associated at least in part with resistance to Hsp90 inhibitors.

It has been known that Hsp90 chaperone activity is regulated by its acetylation status through modulation of the HDAC6-Hsp90 chaperone axis, and acetylation of Hsp90 has been shown to impair the chaperone function of Hsp90 and target its client proteins for degradation [29, 30]. Hsp70 is also an important cochaperone protein of Hsp90 and is required for the assembly of Hsp90-client protein complexes, and acetylation of Hsp70 impaired Hsp90 chaperone activity [25]. In the present study, we found that SIRT1 inhibition promoted the degradation of mut p53 in MDR cells possibly through hyperacetylation of Hsp90/Hsp70 and up-regulation of CHIP in the presence of 17-AAG. Amurensin G also promoted the disruption of Hsp90-p23 interaction. It has been shown that SAHA's inhibition of HDAC6, an essential positive regulator of HSP90, releases mutp53 and enables its MDM2- and CHIP-mediated degradation [31]. Therefore, SIRT1 inhibition appeared to facilitate disruption of Hsp90 chaperone complexes, and increase binding of mut p53 to CHIP. In addition, hyperacetylation of mut p53 by SIRT1 inhibition could contribute to down-regulation of P-gp, since acetylation prevents mut p53 function [32].

It has been reported that P-gp expression in tumor cells may participate in the efflux of Hsp90-directed agents such as 17-AAG, and cause resistance to 17-AAG-mediated anticancer effects [15]. We found that SIRT1 inhibitors inhibited P-gp-mediated efflux in MCF-7/MDR and CEM/VBL_100_ cells via down-regulation of P-gp, suggesting that SIRT1 inhibitor might be effective to increase an efficacy of Hsp90 inhibitors through down-regulation of P-gp and consequently to sensitize MDR cells to Hsp90 inhibitors. Since inhibition of SIRT1 could down-regulate Hsp70/Hsp27 as well as P-gp in various human MDR cancer cells, combinatorial targeting of HSF1/Hsps and P-gp by combined treatment with Hsp90 inhibitor and SIRT1 inhibitor could be a more effective therapeutic approach for Hsp90 inhibitor-resistant MDR cells.

Taken together, the present study suggests that SIRT1 inhibitors would be used to sensitize MDR cells to Hsp90 inhibitors, possibly through suppression of activation of HSF1-dependent pathway and P-gp-mediated efflux activity.

## MATERIALS AND METHODS

### Cell culture and reagents

CEM human lymphoblastic leukemia cell line and its MDR variants, CEM/VLB_55–8_, and CEM/VLB_100_ [33], HeyA8 human ovarian cancer cell line and its MDR subline HeyA8-MDR, MCF-7 human breast cancer cell line and its MDR variant MCF7-MDR (originally named MCF-7/Adr) cells [34] were kindly provided by Dr. Fiedler (MD Anderson, TX, USA). 17-AAG and AUY922 were purchased from Enzo Life Sciences Inc. (Farmingdale, New York, USA) and Selleck Chemicals (Houston, TX, USA), respectively. EX527 was purchased from BioVision Inc. (Milpitas, CA, USA). Amurensin G, a natural SIRT1 inhibitor, was supplied Dr. Oh (Seoul National University, Seoul, Korea) as described previously [35].

### Cell proliferation assay

Cell proliferation was measured by using the 3-(4,5-dimethylthiazol-2-yl)-2,5-diphenyltetrazolium bromide (MTT) assay. Exponentially growing cells (2 × 10^4^ cells/well) were plated in plated in a 96-well plate and incubated in growth medium containing the indicated concentrations of 17-AAG (or AUY922) and/or amurensin G (or EX527) at 37°C for 96 h. Inhibition of cell proliferation was expressed as percentages of untreated control cell growth. At least two separate experiments were performed in triplicate. Interaction between 17-AAG (or AUY922) and amurensin G (or EX527) was assessed using Compu-Syn Software (ComboSyn, Paramus, NJ, USA). A combination index (CI) < 0.9 represents drug synergism, 0.9 < CI < 1.1 implies nearly additive interactions, and CI > 1.1 indicates antagonism. All experiments were carried out in triplicate.

### Western blot and co-immunoprecipitation analysis

Western blot analysis was performed with specific primary antibodies against SIRT1 and CHIP (Cell Signaling Technology, MA, USA), Hsp27 (Epitomics, CA, USA), p53, caspase-3, PARP, Bcl-2, Bax, HSF1, p23, P-gp and pan-acetylated lysine (pan-AcK) (Santa Cruz Biotechnology, CA, USA), Hsp70 and Hsp90 (Enzo Life Sciences, Inc.), and β-actin (Sigma-Aldrich, St. Louis, MO, USA). For co-immunoprecipitation, whole cell extracts from MCF7-MDR cells exposed to 17-AAG or/and amurensin G were incubated with indicated antibody overnight at 4°C. Protein G-Sepharose beads with immunocomplexes were boiled, electrophoresed on 8% SDS-polyacrylamide gels, and analyzed by western blotting was performed using indicated antibody.

### siRNA transfection

The siRNAs were used for the targeted silencing of HSF1 (sc-36511, Santa Cruz Biotechnology, CA, USA), SIRT1 (5′-CUAAUCUAGACCAAAGAAUdTdT-3′) and scrambled control (5′-CUUCCCGAAAACUUGAG ACdTdT-3′) that were purchased from Bioneer (Daejeon, Korea). In brief, MCF7-MDR (or CEM/VLB_100_) cells (2×10^5^ cells/ml) were seeded on 6-well plates and transfected with indicated concentration of SIRT1siRNA or control siRNA using oligofectamine reagent. After 48 h, cells were treated with 17-AAG for an additional 24 h and collected for western blotting to determine the levels of indicated proteins.

### Apoptosis assessment by annexin v staining

MDR cells (2 × 10^5^ cells/ml) were treated with 17-AAG (1 or 10 μM) in the presence or absence of amurensin G (5 μg/ml) for 24 h. Then cells were centrifuged and resuspended in 100 μl of the staining solution containing Annexin V-fluorescein (FITC Apoptosis detection kit; BD ParMingen San Diego, CA, USA) and propidium iodide (Sigma-Aldrich, St. Louis, MO, USA) in in a Hepes buffer. After incubation at room temperature for 20 min, the percentage of early and late apoptotic cells was quantified by FACS for Annexin-V and PI staining.

### Flow-cytometric dye-efflux assay

Cell suspension (500 μl) of MCF7-MDR (or CEM/VLB_100_) cells treated with 17-AAG (AUY922) and/or amurensin G (EX527) for 24 h incubated with 0.5 μg/ml rhodamine 123 (Rho123), a fluorescent substrate of P-gp, at 37°C for 30 min. After incubation, the cells were washed with ice-cold PBS and further incubated at 37°C for 3 h to allow P-gp-mediated drug efflux. Cells were pelleted by centrifugation at 500 × g and resuspended in PBS. Cellular fluorescence was analyzed immediately by using a FACS flow cytometer (FACScalibur, BD Biosciences, San Jose, CA).

### Statistical analysis

The results obtained were expressed as the mean ± S.E. of at least three independent experiments. A Student's *t*-test was used to calculate the statistical significance of the experimental data and the level of significance was set as *p < 0.05, **p < 0.01 and ***p < 0.001.
